# Growth, Development and Temporal Variation in the Onset of Six *Chironex fleckeri* Medusae Seasons: A Contribution to Understanding Jellyfish Ecology

**DOI:** 10.1371/journal.pone.0031277

**Published:** 2012-02-27

**Authors:** Matthew Gordon, Jamie Seymour

**Affiliations:** School of Marine and Tropical Biology, James Cook University, Cairns, Queensland, Australia; Argonne National Laboratory, United States of America

## Abstract

Despite the worldwide distribution, toxicity and commercial, industrial and medical impacts jellyfish present, many aspects of their ecology remain poorly understood. Quantified here are important ecological parameters of *Chironex fleckeri* medusae, contributing not only to the understanding of an understudied taxon, the cubozoa, but also to the broader understanding of jellyfish ecology. *C. fleckeri* medusae were collected across seven seasons (1999, 2000, 2003, 2005–07 and 2010), with growth rates, temporal variation in the medusae season onset and differences in population structure between estuarine and coastal habitats quantified. With a mean of 2 September ±2 d (mean ±95% confidence limits), the earliest date of metamorphosis was temporally constrained between seasons, varying by only 7 d (30 August to 5 September). Juvenile medusae appeared to be added over an extended period, suggesting polyp metamorphosis was an ongoing process once it commenced. At a maximum of 3±0.2 mm d^−1^ IPD, medusae growth to an asymptotic size of ∼190 mm IPD was rapid, yet, with the oldest medusae estimated to be ∼78 d in age, medusae did not appear to accumulate along the coastline. Furthermore, a greater proportion of juveniles were observed along the coastline, with estuarine populations typified by larger medusae. With key aspects of *C. fleckeri*'s ecology now quantified, medusae season management protocols can be further developed.

## Introduction

The occurrence of jellyfish, particularly in blooms, negatively affects a range of recreational, industrial and commercial activities. For instance, while some commercially important fisheries have been unable to function when jellyfish have clogged fishing nets [1], [2], in other fisheries, jellyfish have become predators of and competitors to those species being targeted [2], [3]. Serious industrial issues have also been attributed to increased jellyfish abundances, with power station shut down necessary when water intake pipes have become clogged with medusae [4], [5], [6]. For other regions, it is the medical liability that jellyfish represent that continues to adversely affect the tourism industry which is often integral to local and regional economies [7]. Despite these significant issues, quantitative data documenting key aspects of jellyfish ecology are, in general, lacking. As a result, determining whether claims of increased season length or intensity and frequency of blooms are difficult to validate. For the Australian tropics, it is the seasonal occurrence of *Chironex fleckeri* Southcott that significantly impacts the way in which coastal areas are utilised, yet many of the currently favoured theories relating to the temporal variation in medusae occurrence, medusae growth and development as well as population structure are based on sting records or qualitative data.

The generalisation that *C. fleckeri's* life cycle is seasonal is based on numerous reports of medusae and stings from the warmer months of the year [8], [9], [10], [11], [12], [13], [14], [15], [16], [17], [18] contrasted with a lack of such reports from the winter months. Although the life history of cubozoans is complex [19] in which an asexually reproducing polyp phase alternates with a sexually reproducing medusae phase [15], [16], [20], [21], [22], [23], [24], the timing of the shift from the polyp to medusae phase appears to vary both spatially and temporally [10], [12], [15], [16], [18]. For instance, while *C. fleckeri* medusae typically appear along the far north Queensland coastline in December, they have been reported as early as October in some seasons [12], [15], [18]. There is also a suggestion that the season commences earliest on the west coast, shortly after the first rains of the wet season, with the onset of the stinger season delayed if the wet season is late [10], [25]. Other authors discount the relevance of the wet season [8], [26], however, suggesting that medusae arrival is associated with rising water temperature [10], [16], [26], [27]. With the polyp habitat thought to be located some distance from the coastline within estuary systems [16], the onset of the stinger season is unlikely to accurately reflect the timing of polyp metamorphosis given that the colloquial term ‘stinger season’ typically refers to the arrival of medusae along the coastline rather than the timing of polyp metamorphosis.

Few extrapolations can be drawn from other cubozoan species either, with studies identifying a species cue for metamorphosis limited to a handful of species. Here, increasing water temperature [28], an interaction between increasing water temperature and food [29], [30] or increasing water temperature and photoperiod [31] have been identified as cues. While the development to a specific number of tentacles [32] and the presence of photosymbiotic algae [33] have also been suggested as potential cues for metamorphosis, these links have not been validated quantitatively. Similarly, while the correlation between rainfall events and successive pulses of juvenile cubomedusae [34], [35] may implicate salinity as a cue for metamorphosis, quantitative data demonstrating this link is lacking. Given this paucity in data, a need therefore exists for research distinguishing between those mechanisms driving polyp metamorphosis and those merely correlated with this process.

At metamorphosis, juvenile *C. fleckeri* medusae are approximately 1.2 to 1.4 mm in size [24], but increase in size rapidly [15], [16], [17], reaching sexual maturity late in the season [10], [15]. While such growth patterns are reported for a number of scyphozoan species [36], for cubomedusae, it is only for *Chiropsella bronzei* Gershwin that growth parameters have been quantified [34], [35]. This paucity in data is largely due to the lack of a reliable method by which cubomedusae can be aged. Size of cubomedusae, for instance, is an unreliable indicator of age, given that degrowth of the bell can occur in cases where feeding regimes are inadequate (for example, an underfed captive *C. fleckeri* medusa [37]). While some authors have used the number of tentacles per pedalium as an indicator of development [25], tentacles are added across a size range, and hence, may still be somewhat dependent upon feeding regime. More recently, the statoliths contained within the statocysts of cubomedusae rhopalia (eye bearing sensory structures) have been shown to contain fine growth rings that are added on a daily basis [35], [38], [39], [40], [41].

Insights into the ecology of a species can also be gained from population structure data. For example, several cohorts of *C. bronzei* medusae occurring within a single season, as well as a correlation between cohort appearance and significant rainfall events, were elucidated from population structure data [34], [35]. Comparable data for *C. fleckeri* populations is, however, currently limited to the generalisation that an abundance of small individuals early in the season progresses to fewer but larger medusae late in the season [15]. Given that the shift from estuarine to coastal habitats is thought to accompany the shift from polyp to medusae phases of the life cycle [16] whereby medusae are washed from within estuaries at the onset of the season [15], [42], coastal populations are likely to be dominated by larger and older medusa, while smaller and younger medusae would typify estuarine populations. Yet, it would appear that large medusae can be present even in early season samples [10], [14] and “no specific rule can be given to the range of sizes encountered in a given area at a specific time” [10]. Whether the presence of only large medusae at a given location and small medusae in another indicates the presence of a nursery location [14], or perhaps the polyp habitat itself, is yet to be demonstrated. A need therefore exists to quantify aspects of population structure if the overall ecology of *C. fleckeri* is to be better understood.

The current paper aims to quantify some of the long held theories relating to the ecology of *C. fleckeri* medusae. Key aspects to be investigated include the:

○ relationship between statolith size and growth rings
○ temporal variability in the onset of medusae production (metamorphosis)
○ growth and development of *C. fleckeri* medusae
○ population structure of coastal and estuarine medusae populations.

## Methods

### Sample Sites

**Figure 1:**
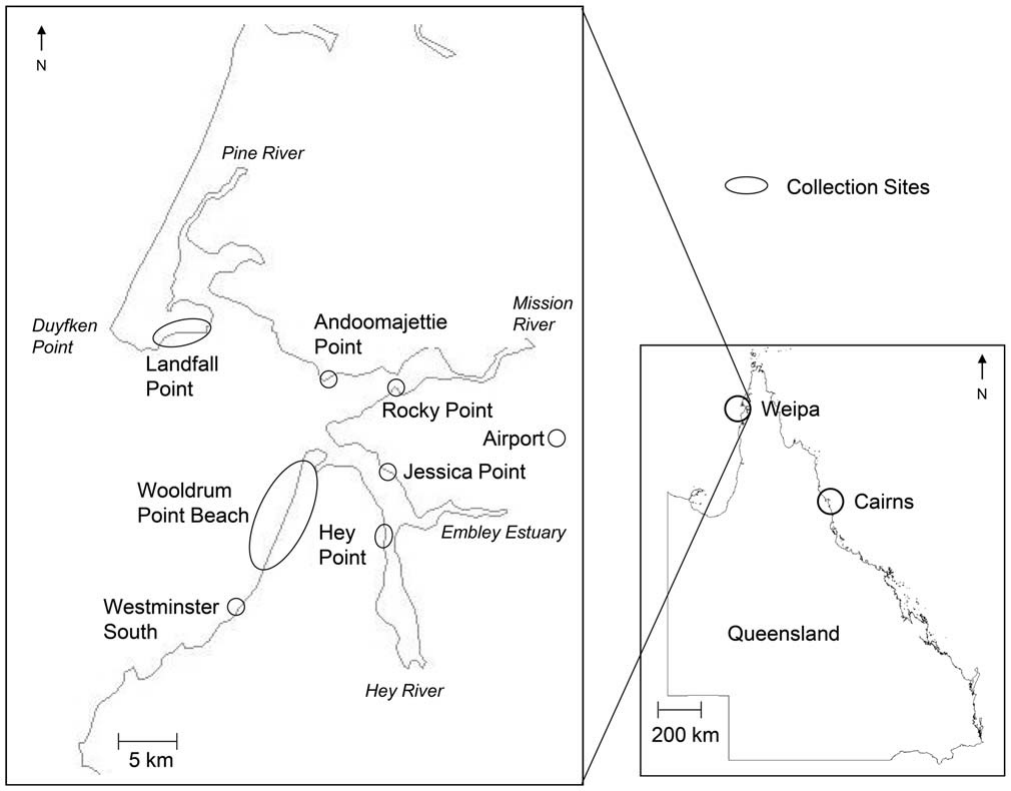
Geographic location of Weipa medusae collection sites, western Cape York, north Queensland, Australia.

A total of 484 medusae were collected from seven sites at Weipa during the 1999, 2000, 2003, 2005, 2006, 2007 and 2010 stinger seasons. While not all sites were visited on each occasion or within each season, Landfall Point (12°34′53″S, 141°39′50″E), Andoomajetti Point (12°36′20″S, 141°49′21″E), Rocky Point (12°37′10″S, 141°52′38″E), Jessica Point (12°40′05″S, 141°51′42″E), Hey Point(12°44′23″S, 141°53′35″E), Wooldrum Point Beach (12°42′19″S, 141°48′03″E) and Westminster South (12°50′11″S 141°44′56″E) represented Weipa (Figure 1). A further 46 medusae were collected during the 2005, 2006 and 2007 seasons from 12 east coast sites between Cairns and Townsville, which included Mission Beach, Gin Camp, Yorkeys Knob, Port Douglas, Cardwell, Palm Cove, Buchan Point, Lugger Bay, Townsville and the Tully, Murray and Hull Rivers. Given the low numbers collected on each occasion, east coast sites were excluded from most analyses.

Weipa sites were classified as coastal or estuarine sites. Coastal sites were those that occurred along a beachfront or the embayment of Albatross Bay, such as Wooldrum Point Beach. Estuarine sites were those that occurred wholly within an estuary system or at the intersection of an estuary system and the coastal embayment of Albatross Bay, such as Andoomajetti Point.

### Statolith Technique

Medusae were collected by hand and their inter pedalia distance (IPD), the distance between the mid line of alternate pedalia along the line passing through the rhopalia, was measured to the nearest mm. Each of the four rhopalial niches and a gonad sample (if gonads were evident) were removed and preserved in 98% ethanol.

For 71 medusae, the statoliths of two rhopalia were dissected from the base of their eye set. Undamaged and unshattered statoliths were cleaned of any cellular material before being embedded in resin in the profile plane (kidney shape was evident). The top 50% of each statolith was ground using 1200 gauge wet and dry sand paper, polished with Brasso, rinsed and then polished with tooth paste. The number of rings present in each statolith was counted under oil immersion using ×400 magnification on a light microscope. Each dark band with a light band either side was considered to be one growth ring. The length of each statolith was measured using a calibrated stereo dissector microscope, with length taken to be the longest distance between curved apical ends of a statolith. Both the average number of rings and average length of each statolith pair were then calculated, with the relationship between the number of growth rings and statolith length investigated using regression analysis.

Two statoliths from a further 437 medusae were dissected and their length measured under a calibrated stereo dissector microscope. The average length of each statolith pair was calculated, from which, the number of rings was estimated using the relationship between statolith length and number of rings.

### Quantifying the Time Frame of Statolith Growth Rings

Establishing a significant relationship between medusae size (mm IPD) and age (number of rings in statolith) was a two stage process. Firstly, regression analysis was used to determine whether medusae size (IPD mm) could be predicted from tentacle number, and secondly, whether tentacle number was correlated with ring number. Given that medusae can undergo both growth and degrowth, tentacle number was considered a more reliable indicator of medusae development as tentacles are not lost once added.

To be able to age medusae, however, it was necessary to quantify the unit of time represented by successive growth rings. While tetracycline is widely used for growth ring time frame validation in fish otoliths, several attempts at applying this technique to *C. fleckeri* medusae were unsuccessful, despite various concentrations of tetracycline being trialled and medusae being housed in large, custom made cylindrical tanks. While this has limited the methods by which the interval between successive rings can be quantified, several pieces of evidence suggest that successive rings are added on a daily basis. Firstly, if growth rings were added at hourly or weekly intervals, not only are these arbitrary units of time that medusae would be unable to measure, but would also make medusae within this study less than four days (hourly) or 1.5 years (weekly) old. Monthly or annual units of time are also unrealistic given that the oldest medusae would have been 7 or 80 yrs of age respectively. Rather, if successive rings were added on a daily basis, medusae collected within this study would have ranged between one and three months of age. Not only are these realistic age estimates, given that medusae are unlikely to survive between seasons, but growth rings are added on a daily basis in three other species of cubozoa [35], [38], [39], [40], [41]. In this scenario, alternating dark and light bands would reflect the diurnal behaviours of medusae [25], [43]. That is, throughout the day, medusae expend considerable amounts of energy swimming and feeding [15], with less energy available for growth. In contrast, reduced activity levels at night [43] would allow relatively more energy to be devoted to growth. Under this scenario, alternating light and dark bands would arise from the variation in statolith density associated with differential growth between day and night. Collectively then, the most plausible unit of time between consecutive growth rings is daily, as has been shown in other cubozoans [35], [38], [39], [40], [41].

### Calculating Date of Metamorphosis

Only medusae collected from Weipa sites were included in these analyses, as small sample sizes from east coast sites made analysis unreliable. The age of 461 medusae was taken to be either (a) the number of rings observed within their statoliths (64 medusae) or (b) the number of rings estimated from the relationship between statolith length and ring number (397 medusae). The metamorphosis date of each individual was calculated by subtracting an individual's age from its date of capture. The percentage of each sample that underwent metamorphosis on a given day was calculated and plotted against season number. Season number was used in preference to year number as it allowed successive samples within a season to be plotted together. That is, a sample collected in January 2007 was denoted a season number of 2006 as was a sample collected in November 2006.

Temporal variation in the onset of the 2000, 2003, 2005, 2006, 2007 and 2010 seasons was quantified by calculating the average earliest metamorphosis date of these six seasons and the associated 95% confidence limits.

A number of environmental parameters are potentially relevant to the shift from the polyp to medusae phase of the life cycle, with the following parameters quantified for the five weeks prior to the onset of metamorphosis in each season:

○ water temperature was taken to be the daily sea surface temperature at midday at 16°35′23″S, 141°33′36″E (Albatross Bay) and was obtained from the Integrated Marine Observing System at www.marine.csiro.au/remotesensing/imos
○ daily rainfall totals were obtained from the Bureau of Meteorology for Weipa Eastern Avenue (location 27042).
○ photoperiod, or the total number of hours of daylight per day, was calculated from sunrise and sunset times for Weipa obtained from Geoscience Australia at http://www.ga.gov.au/geodesy/astro/sunrise.jsp
○ tidal amplitude was calculated from hourly tide height (m) data for Humbug Wharf, obtained from Maritime Safety Qld at http://www.msq.qld.gov.au/Home/Tides.

Data were assigned to a week category (1 to 5) in which week category represented the number of weeks prior to the onset of metamorphosis for that season. The weekly variation between years in each of these parameters was investigated using a two way Analysis of Variance in which both week category and year were fixed factors. The date of the full moon within the five weeks prior to the onset of metamorphosis in each season was obtained from Geoscience Australia at http://www.ga.gov.au/earth-monitoring/geodesy.

### Growth Curve Calculations

Gordon *et al.* [35] established that a Gompertz growth equation most accurately described the growth parameters of a closely related cubozoan, *C. bronzei*, using the criteria described by Kauffman [44]. Parameters for a four criteria Gompertz growth curve were estimated in Sigma Plot 10 using size at age data for 461 medusae. Size was taken to be IPD at time of capture and age was either (a) the number of rings present in an individual's statolith (64 medusae) or (b) an estimate using the relationship between statolith size and ring number (397 medusae). The maximum daily growth rate (mm d^−1^) and 95% confidence limits were estimated by regression analysis of the linear component of the growth curve, which occurred between 40 and 70 d. The time to the onset of sexual maturity was also estimated using the resulting growth equation.

### Investigation of Population Structure

Since each site was not visited on each occasion, data for medusae collected in Weipa were pooled for all seasons and sites within habitat type. East coast sites were not analysed here due to small sample sizes. Those medusae in which gonads had not developed were denoted as immature juveniles. For mature individuals, sex was determined by examining gonad samples under a stereo dissecting microscope. Individuals in which ova were visible were denoted as females, while those in which sheets of convoluted filamentous tissue were evident were denoted as males. In those cases where it was unclear whether tissue was that of a small male or an immature specimen, individuals were classified as undistinguishable (∼60 individuals) and grouped with immature specimens. A chi squared homogeneity test was used to determine whether any significant difference between the proportion of males, females and juveniles existed between the estuarine and coastal habitats.

## Results

**Figure 2:**
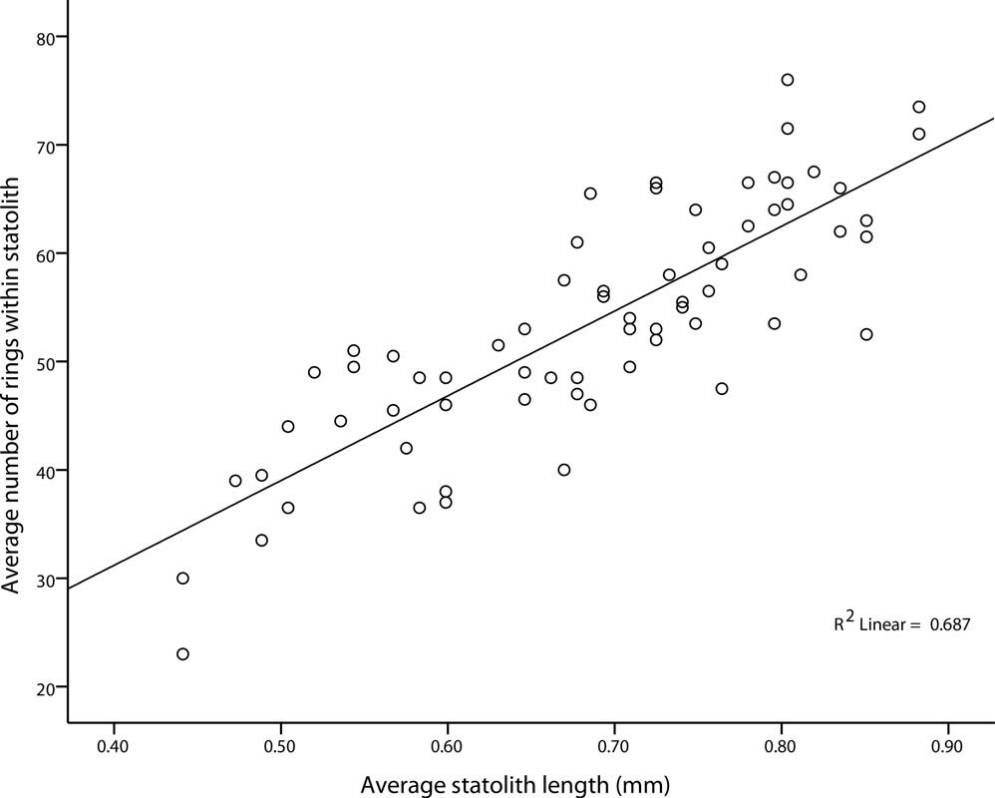
Positive linear relationship between statolith length and number of rings within a *Chironex fleckeri* statolith. Statolith length is in mm and number of rings is averaged for statolith pairs.

A significant and positive relationship between statolith length (mm) and number of rings was established (F = 151.243, df = 1×69, n = 71, *P*<0.001, R^2^ = 0.687), whereby the number of rings present within a statolith increased as did statolith length. Within the size range of statoliths sampled, a linear relationship (Figure 2) provided a better fit than curvilinear equations, with this linear relationship best described by the equation:
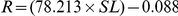
where *R* is the number of rings within a statolith and *SL* is statolith length in mm.

**Figure 3:**
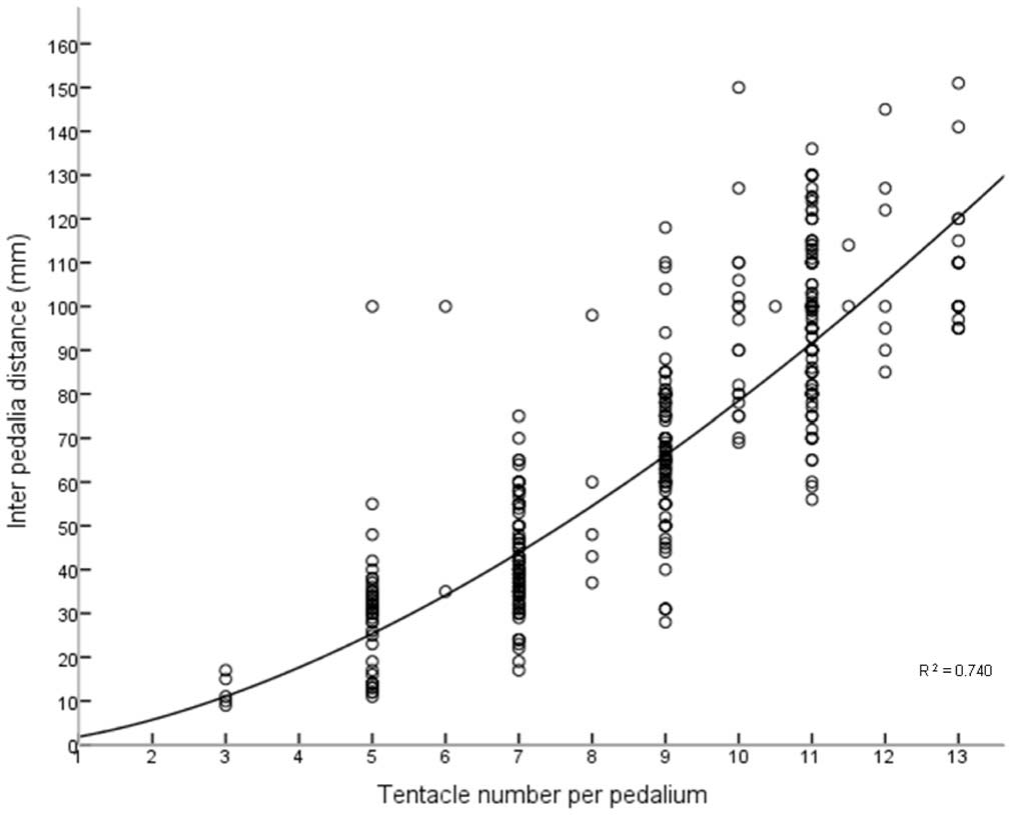
Positive curvelinear relationship between number of tentacles and Inter Pedalia Distance for *Chironex fleckeri* medusae. Inter pedalia distance is in mm and tentacle number is per pedalium.

A significant and positive relationship was established between the number of tentacles per pedalium and inter pedalia distance (mm) (F = 1201.176, df = 1×420, n = 422, *P*<0.001, R^2^ = 0.740) whereby medusae size (IPD in mm) increased as did the number of tentacles per pedalium (Figure 3). A power curve described this relationship most appropriately, with inter pedalia distance increasing at a faster rate as more tentacles were added to each pedalium. This curve provided a minimum medusae size at the one tentacle stage (ie following metamorphosis) of ∼1.8 mm and is best described by the equation:
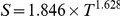
where *S* is medusa size (IPD) in mm and *T* is the number of tentacles per pedalium.

**Figure 4:**
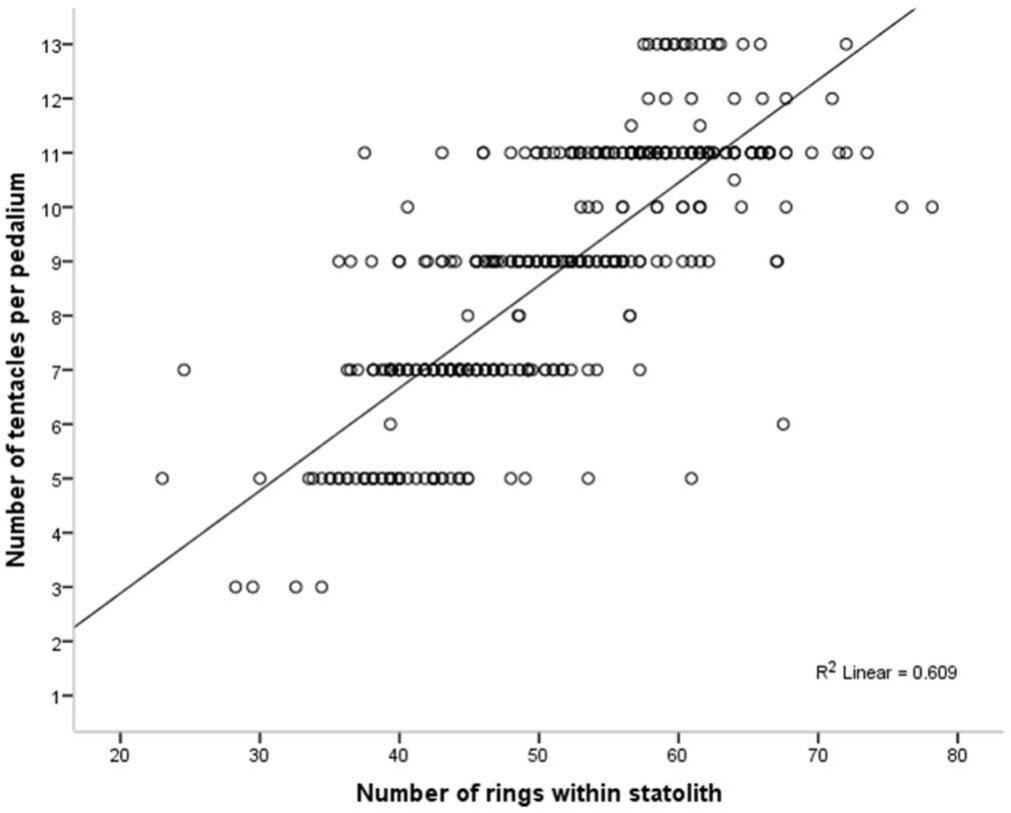
Positive linear relationship between number of rings and number of tentacles for *Chironex fleckeri* medusae. Number of rings is the average per statolith and tentacle number is per pedalium.

Medusae appeared to add tentacles in pairs, with only ∼8% of the 422 medusae for which tentacle number was collected possessing an even number of tentacles per pedalium. A significant and positive relationship was identified between the number of rings within a statolith and the number of tentacles suspended from each pedalium (F = 639.733, df = 1×410, n = 412, *P*<0.001, R^2^ = 0.609). Within the age range of medusae sampled, a linear relationship in which tentacle number increased as did ring number (Figure 4) provided a better fit than did curvilinear equations and is best described by the equation:
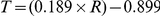
where *T* is the number of tentacles per pedalium and *R* is the number of rings per statolith.

The earliest date of metamorphosis was 30 August and occurred in the 2007 season. Despite the earliest date of specimen collection varying by 33 d, the earliest date of metamorphosis did not vary by more than 7 d. That is, while medusae were first collected on the 18^th^ October in the 2000 season in which the earliest date of metamorphosis was 31 August, in the 2010 season for which the earliest metamorphosis date was 5 September, medusae were not collected until the 20^th^ November. The mean earliest date of metamorphosis was 2 September ±2 d (mean ±95% confidence limits).

**Figure 5:**
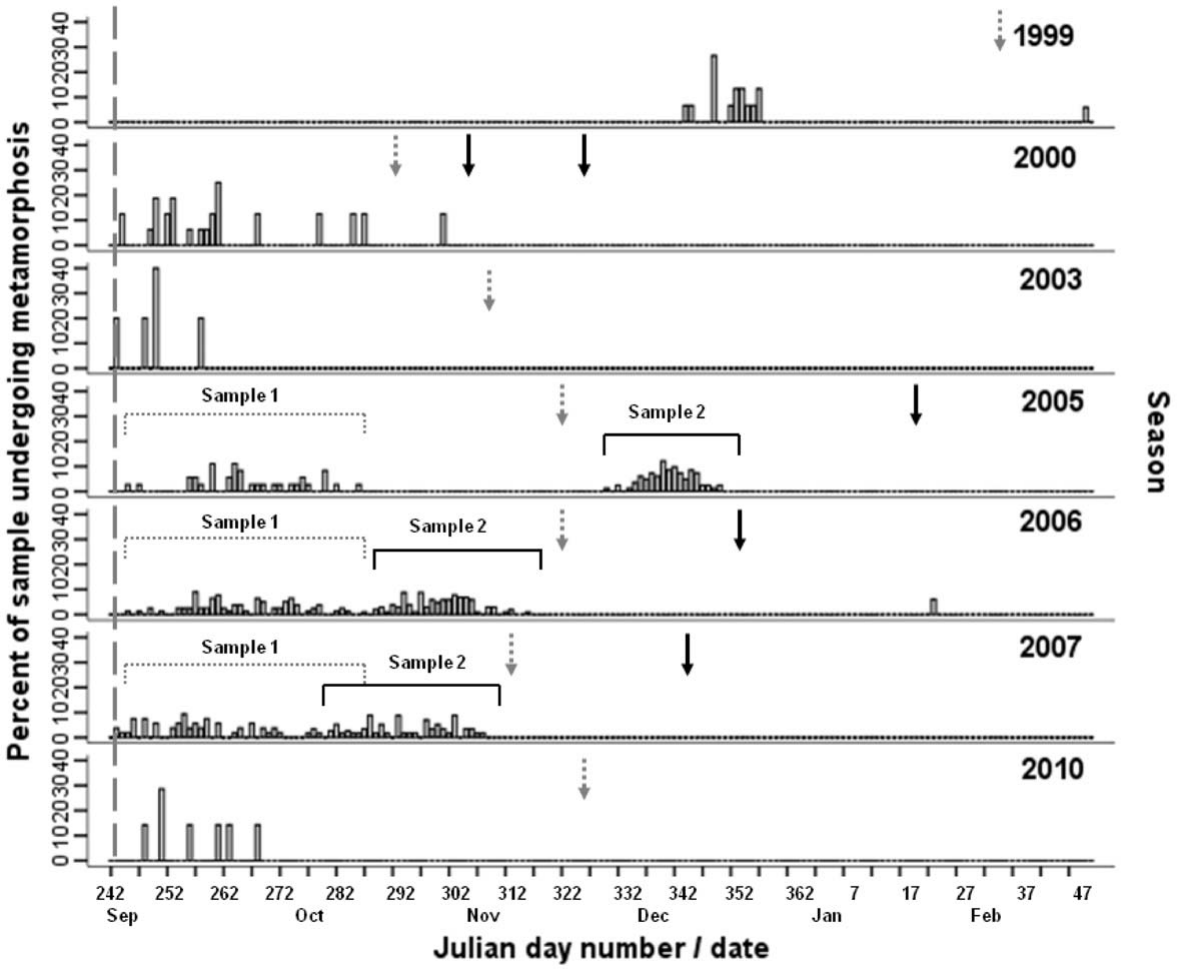
Percent frequency of *Chironex fleckeri* medusae that metamorphosed each day. Frequency is percentage within a sample and date is Julian day number or month, with 30 August referenced by a grey dash line. The first sampling occasion within a season is denoated by a grey arrowhead line, while subsequent sampling occasion(s) are denoted by a black arrowhead line.

Within the 2005, 2006 and 2007 seasons in which sample sizes were large, medusae were added to the population on an almost daily basis, with each date of metamorphosis represented by ∼2%, with no one date accounting for more than 10% of a sample (Figure 5). Metamorphosis also appeared to be an ongoing process once it commenced. That is, in those seasons where the interval between successive samples (between grey and black arrowhead lines) was approximately one month, as for the 2006 and 2007 seasons, metamorphosis dates were either continuous (2006 season) or overlapped by a small amount (2007 season). In the 2005 season, however, successive samples were collected ∼60 d apart (November 2005 and January 2006), with the gap in metamorphosis corresponding to the length of time between sampling occasions.

Water temperature (°C), daily rainfall (mm) and tidal amplitude (m) each showed significant weekly variation between years in the five weeks prior to the earliest date of metamorphosis within each season (Table 1). Photoperiod (h daylight d^−1^), however, did not vary significantly by week between years (Table 1). The dates of the full moon in the five weeks preceding the onset of metamorphosis differed by 19 d across the six seasons studied here, ranging from 9 August in 2006 to 28 August in 2008.

**Table 1:** ANOVA results for the variation in parameters potentially associated with metamorphosis.

Parameter	Interaction	F	df	*P*
Water Temperature (°C)	week category×year	2.031	20×192	0.008
Total daily rainfall (mm)	week category×year	1.814	20×210	0.022
Tidal amplitude (m)	week category×year	1.696	10×697	0.030
Photoperiod (h daylight d^−1^)	week category×year	0.088	20×210	1.000

Water temperature is in °C, total daily rainfall is in mm, tidal amplitude is in m and photoperiod is the number of h of daylight d^−1^ for each week in the five weeks prior to the onset of metamorphosis in each season.

**Figure 6:**
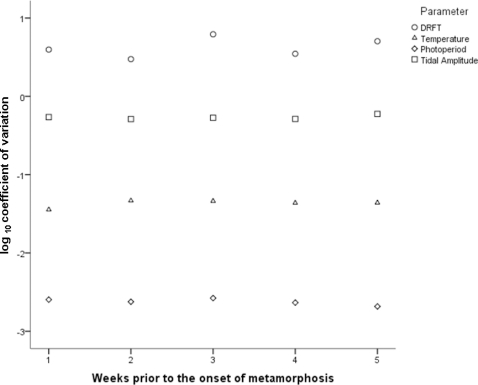
Coefficient of variation (log_10_ transformed) of environmental parameters. CV for daily rainfall total (mm), tidal amplitude (m), water temperature (°C) and photoperiod (h daylight d^−1^) for the five weeks preceding the earliest date of metamorphosis in each season.

The degree to which each environmental parameter varied in the five weeks prior to the onset of metamorphosis was parameter specific. That is, daily rainfall total (DRFT in mm) showed the greatest degree of variation, having a log_10_ transformed CV value of ∼1, while photoperiod showed the least amount of variation with a log_10_ transformed CV value of ∼−3 (Figure 6). The variation in both water temperature (°C) and tidal amplitude (m) was also considerably greater than photoperiod, ranging from ∼0 (tidal amplitude in m) to ∼−1.5 for water temperature (°C) (Figure 6).

**Figure 7:**
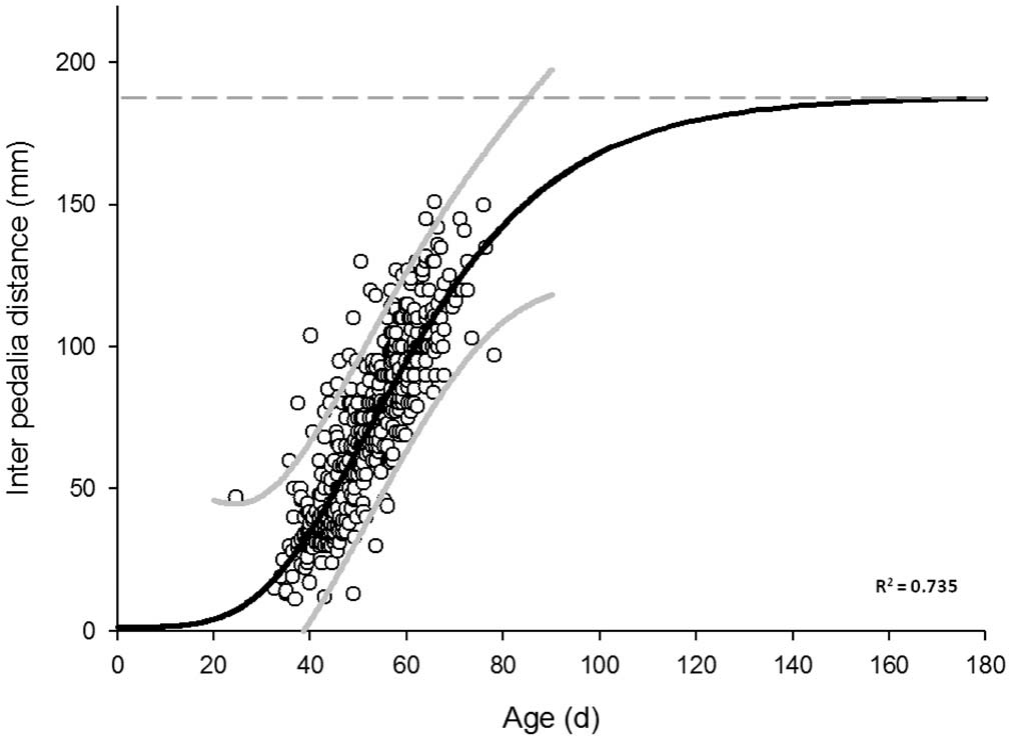
Gompertz growth curve for significant, positive relationship between age and IPD of *Chironex fleckeri* medusae. Growth curve shows medusae size (IPD) in mm at age in d with 95% prediction limits (grey solid line).

A significant and positive relationship between IPD (mm) and age (days) of medusae (F = 423.3479, df = 3×457, n = 461, *P*<0.0001, R^2^ = 0.735) whereby medusae increased in size with age towards an asymptotic size of ∼190 mm IPD (Figure 7). The Gompertz growth equation had the following format:
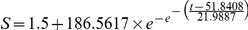
where *S* is medusa size (IPD) in mm and *t* is medusa age in days.

Regression analysis of the linear component of this relationship (from 40 to 70 d) revealed a maximum growth rate of ∼3 mm d^−1^ (±0.2 mm d^−1^). The minimum size (IPD in mm) at which males and females could be reliably distinguished was 46 mm IPD and 50 mm IPD respectively. According to the above growth equation, it would take ∼45 d to reach the average size (IPD mm) of sexual differentiation. At an average 96 mm IPD for male medusae and 97 mm IPD for females, the mean size of male and female medusae did not vary significantly between the sexes (F = 0.25, df = 1×240, n = 242, *P* = 0.621).

**Figure 8:**
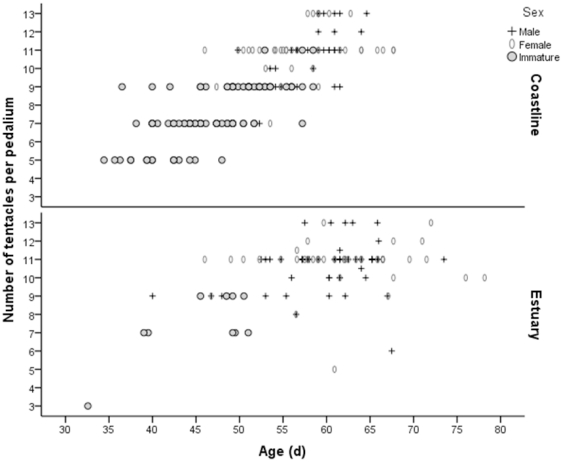
Age of *Chironex fleckeri* medusae with respect to tentacle number. Distinction is made between males, females and immature medusae within the coastal and estuarine habitats of Weipa, where age is in d and tentacle number is per pedalium.

The age structure of the overall population differed significantly between the estuarine and coastal habitats (χ^2^ = 49.477, df = 2, n = 281, *P*<0.001). Younger medusae were not as well represented within the estuarine habitat, with a predominance of larger and older individuals evident instead. Along the coastline, however, a greater spread of males, females and immature medusae was observed, ranging in age from 30–70 d and from five to 13 tentacles per pedalium (Figure 8). Furthermore, the oldest medusae within the estuarine habitat were older than those along the coastline (Figure 8). Although medusae added new tentacles over an age range, it was typically possible to determine the sex of an individual providing it possessed more than nine tentacles. This was consistent between the estuarine and coastal populations for all but three medusae within the coastal habitat who were classed as immature at the 11 tentacle stage.

## Discussion

One area of jellyfish ecology that has received increasing attention of late is the shift from the polyp to the medusae phase, and the factors associated with, or acting as cues for this shift. Not only was the onset of medusae production temporally constrained between seasons, but it also commenced earlier than expected. That is, the earliest metamorphosis date was 30 August (2007 season) and varied by only ∼7 d across six seasons. Not only is September also the earliest month in which juvenile medusae are reported in plankton tows from east coast studies [15], but medusae of 120 mm collected in December on Magnetic Island [14] would be ∼70 d of age (from the above growth curve), also giving them a metamorphosis date in September. This result is particularly significant in terms of modelling the overall medusae season in that the onset of each season can now be defined with greater accuracy.

Quantifying when polyp metamorphosis commenced also allows the factors associated with the shift from the polyp to medusae phase to be identified. It is these factors that polyp based studies should include when quantitatively indentifying the cue for metamorphosis. For instance, the significant between year variation in weekly mean water temperature suggests that water temperature is unlikely to have provided the temporal periodicity observed in the onset of metamorphosis within this study. A similar case exists for both tidal amplitude and rainfall. That is, while unusually high amplitude tides could result in salinity changes at the polyp habitat by pushing higher salinity waters further into estuary systems or allowing fresh waters to drain further down estuaries, the timing of any tidally driven salinity fluctuations would have varied between years. Likewise, significant between year variation in total daily rainfall (mm) suggests that rainfall (or rainfall driven salinity changes) was unlikely to have acted as a cue for the onset of polyp metamorphosis in the seasons investigated within this study. Indeed, the climate of Weipa is dominated by strong seasonal patterns [45], [46], [47], with low rainfall and elevated, stable salinity typical for late August/early September [46]. Salinity did not appear to be related to the metamorphosis of *C. fleckeri* polyps in laboratory based trials [15], [24] either, although dilution rates may have induced encystment rather than metamorphosis. The influence of temperature is also unclear from polyp based studies, with all trials conducted at 28°C [15], [24]. While the results of this study suggest that salinity, temperature, tidal amplitude or moon phase are unlikely to provide the temporal consistency in the onset of metamorphosis as was observed within this study, their role as interacting variables cannot, as yet, be disregarded.

One parameter that could provide a higher degree of temporal consistency in the onset of metamorphosis is photoperiod, with the average hours of daylight consistent between seasons in the five weeks prior to the earliest date of metamorphosis within each season. The influence of photoperiod on polyp metamorphosis remains largely untested, however, with *Carybdea morandinii* the only cubozoan for which a link between photoperiod and metamorphosis has been established [31]. Not only has light been positively correlated with asexual reproduction for some Scyphozoans [48], [49], but Purcell [48] suggests that melatonin, a light sensitive hormone, may also play an important role in coordinating the strobilation of the Scyphozoan *Aurelia labiata*. Photoperiod, which has been shown to coordinate breeding cycles in some marine invertebrates [50], may play a similar role in coordinating polyp metamorphosis in *C. fleckeri*, and as such future polyp based research should quantify the significance of photoperiod on polyp metamorphosis.

Metamorphosis did not appear to be a single or pulse event for *C. fleckeri*, rather, an ongoing process whereby low numbers of medusae were produced on an almost daily basis (between 2 and 10% d^−1^). The collection of a medusa in March 2000 (metamorphosis date of February 19) and another in March 2007 (metamorphosis date of January 23) further suggests that medusae production continued over an extended time frame. Reports of 0.6 to 1.8 mm juvenile *C. fleckeri* medusae in estuarine plankton samples between September and January [15], the presence of both adult and small medusae in the first arrivals along the coastline [10], [14], as well as the collection of 6 mm medusae in January and February when 120 mm medusae were collected in December further suggest that metamorphosis occurs over an extended timeframe. Reports of juvenile medusae occurring in successive waves in the only laboratory based study conducted on *C. fleckeri* polyps [24] initially appear contradictory to the results of this study, however, without the timeframe between successive waves quantified, the potential for pulses to have occurred on a daily basis, as observed within this study, cannot be disregarded.

Once within the sexual phase of the life cycle, medusae growth was rapid at up to 3±0.2 mm d^−1^, which is up to three times that established for a closely related and often co occurring species, *C. bronzei* [34], [35]. Although growth rates are likely to vary between individuals due to prey availability [8], [15], medusae would typically reach their estimated asymptotic size of ∼190 mm (IPD in mm) after ∼140 days. That *C. bronzei* has an estimated asymptotic size of 71 mm IPD [34], [35] and *Chiropsalmus quadrumanus* Agassiz is reported to reach 110 mm [51] suggests that *C. fleckeri* medusae attain a larger size than do other Chirodropid species. On an applied level, Hartwick [16] has previously suggested that medusae reach a size dangerous to humans within approximately two to three months. Indeed, *C. fleckeri* medusae undergo an ontogenetic shift in their cnidome (and diet) that potentially explains the lethality of larger medusae to humans [52] at ∼60–100 mm IPD [52], a size they would reach after ∼50–65 d. This is an important parameter to consider in the further development of management protocols in that the time at which medusae are likely to become lethal to humans can now be defined with greater reliability.

That the onset of sexual maturity occurred at approximately ∼50 mm IPD compares favourably to estimates provided by Barnes [10] who noted that the development of a very large area of gonad material commenced at the eight tentacle stage (∼60 mm IPD based on the regression equation developed here) [25]. At the rapid rate of growth quantified here, medusae would become sexually mature after ∼45–50 d, which is considerably less time than the typical length of a season (∼180 d). Given that the oldest medusa was ∼78 d, it does not appear that medusae accumulate as the season progressed [15]. Potentially, medusae relocated from within the estuarine and coastal areas sampled here, with the infrequent collection of medusae up to several km from shore [8], [37], [53] suggesting that some form of emigration could take place. Alternatively, medusae that underwent metamorphosis in early September would have had several months of stable conditions and an abundant food supply prior to the onset of the wet season in which to grow and mature. That is, September to December falls within Weipa's dry/pre wet season [47], [54] when salinity regimes are typically stable and elevated [47], water temperatures are typically increasing [45], [46], [47] and an abundance of post larval prawns occurs within the Embley Estuary [46], [47]. Whether several generations of medusae occur within a single season is an aspect of *C. fleckeri*'s ecology that future research should examine.

Medusae development can also be considered with respect to tentacle number, with some authors using tentacle number rather than size when discussing the development of medusae (e.g. [25]). The maximum number of tentacles per pedalium observed within this study was 13, which compares favourably to maximums of 12 [10], [11] and 13 [25], but suggests that 15 tentacles may be limited to those individuals of ∼300 mm IPD which are rarely observed [8], [12], [55]. Given that newly metamorphosed medusae possess one tentacle per pedalium [24] and only 8.3% of medusae within this study possessed an even number of tentacles, *C. fleckeri* would appear to add tentacles in pairs. With 12 medusae possessing an odd number of tentacles and 10 possessing a even number of tentacles, samples in Kinsey [25] suggest that tentacles are added singularly, however this may be an artefact of a small sample size. *C. bronzei* also appears to add tentacles in pairs, with 92% of the 1652 medusae collected possessing an odd number of tentacles [34]. On a more applied level, tentacle number may provide a more standardised method by which groups such as Surf Life Saving can provide consistent estimates of medusae size and age, given the significant relationships that exist between these variables.

A difference in the population structure of coastal and estuarine habitats would be expected if a seasonal alternation in generations and habitats [16] is occurring for *C. fleckeri*. That is, juvenile medusae would be representative of estuarine populations while coastal populations would be typified by both a greater range in medusae size as well as an accumulation of larger individuals as the season progressed. Not only were medusae from coastal sites typically smaller (fewer tentacles) and younger than those found within the estuarine habitat, but the oldest medusae (∼78 d) was also collected from within the estuarine habitat. While these results appear contradictory to expected patterns, juvenile medusae reported by Hartwick [15] were as small as 0.6 mm and collected in plankton tows [15], [16], with the visually based collection techniques used within this study possibly possessing an inherent bias against such small individuals. Differences in population structure may also reflect the suitability and availability of prey within the estuarine and coastal habitats. For instance, not only have mangrove areas of the Embley Estuary been shown to be important nursery areas for many species [56], but a greater abundance of fish [54] and prawn species [57] have been reported for intertidal areas adjacent to mangrove stands. In contrast, both species diversity and overall abundance of fish was lower along the coastline [58]. Further data quantifying medusae abundance, gastrovascular cavity content and prey abundance is required to further validate these claims.

Collectively, the results of this study are relevant in both an applied and an ecological context. By quantifying growth and development rates as well as the temporal variation in the onset of polyp metamorphosis between seasons for *C. fleckeri* medusae, this paper has contributed to the understanding of the ecology of an understudied taxon, the cubozoa, as well as to the broader understanding of jellyfish ecology. On an applied level, this study has presented quantitative data upon which models predicting the seasonal occurrence of this species can be developed. For instance, estimates of when medusae are likely to present a considerable risk to humans can now be based on medusae growth and development rates, allowing significant events, such as the ontogenetic shift in cnidome, to be modelled with greater accuracy. It is in this way that the negative effects of the stinger season can be managed more effectively. Such models are of particular relevance given the way in which the seasonal occurrence of *C. fleckeri* impacts the way in which the tropical Australian coastline is utilised throughout the warmer months of the year. However, it is only when a complete understanding of the medusae phases ecological relationships are developed that the occurrence and distribution of *C. fleckeri* can be modelled with accuracy and reliability. This study represents the first attempt at quantifying such parameters, however, further long term studies are required if management practices are to be optimised and broader ecological questions regarding season length or the intensity and frequency of jellyfish blooms are to be validated.
